# Adjunctive efficacy of *Bifidobacterium animalis* subsp. *lactis* XLTG11 for functional constipation in children

**DOI:** 10.1007/s42770-024-01276-3

**Published:** 2024-02-21

**Authors:** Ke Chen, Zengyuan Zhou, Yang Nie, Yanmei Cao, Ping Yang, Ying Zhang, Ping Xu, Qinghua Yu, Yang Shen, Weiwei Ma, Shanshan Jin, Changqi Liu

**Affiliations:** 1 Department of Nutrition, School of Medicine, Chengdu Women’s & Children’s Central Hospital, University of Electronic Science and Technology of China, Chengdu, China; 2 Department of Child Health Care, Chongzhou Maternal and Child Health Care Hospital, Chengdu, China; 3 Department of Child Health Care, Dayi Maternal and Child Health Care Hospital, Chengdu, China; 4 Department of Child Health Care, Xindu Maternal and Child Health Care Hospital, Chengdu, China; 5 Department of Child Health Care, Jinniu Maternal and Child Health Care Hospital, Chengdu, China; 6 Department of Child Health Care, Qingbaijiang Maternal and Child Health Care Hospital, Chengdu, China; 7 Laboratory of Microbiology, Immunology and Metabolism, Diprobio (Shanghai) Co, Limited, Shanghai, China; 8 College of Pharmacy, Heilongjiang University of Chinese Medicine, Harbin, China; 9 School of Exercise and Nutritional Sciences, San Diego State University, San Diego, USA

**Keywords:** Probiotics, Constipation, Children, Gut microbiota

## Abstract

Functional constipation (FC) can seriously affect the physical and mental health of children. The goal of this study is to assess the efficacy and safety of *Bifidobacterium animalis* subsp. *lactis* XLTG11 in treating FC in children through a randomized, double-blinded, placebo-controlled approach. Eligible children were randomized into either the intervention group (IG, *n* = 65, receiving conventional treatment with probiotics) or the control group (CG, *n* = 66, receiving conventional treatment without probiotics). The primary outcome measure was fecal frequency. Fecal gut microbiota analysis and PICRUSt (Phylogenetic Investigation of Communities by Reconstruction of Unobserved States) were used to predict gene family abundances based on 16S information. Over the course of treatment, the weekly frequency of feces within each group increased significantly (*F* = 41.97, *p* < 0.001). The frequency of feces (times/week (t/w)) in the IG was significantly higher than that in the CG (3.69 ± 2.62 t/w vs.3.18 ± 1.43 t/w, 4.03 ± 2.54 t/w vs. 2.89 ± 1.39 t/w and 3.74 ± 2.36 t/w vs. 2.94 ± 1.18 t/w and 3.45 ± 1.98 vs. 3.17 ± 1.41 t/w for the 1st, 2nd, 3rd, and 4th week after intervention, respectively) (*F* = 7.60, *p* = 0.0067). After the intervention, dominate species shifted to *Bifidobacterium longum*, *Bifidobacterium breve*, and *Escherichia coli* in the IG. Additionally, genes related to short-chain fatty acid (SCF) metabolism were upregulated, while methane metabolism was downregulated. Administration of XLTG11 at a dose of 1 × 10^10^ CFU/day to children increased fecal frequency, induced beneficial changes in gut microbiota, and regulated SCFs and methane metabolism–related genes.

**Supplementary Information:**

The online version contains supplementary material available at 10.1007/s42770-024-01276-3.

## Background

The incidence of infant constipation is influenced by many factors, and the reported incidence rate varies in China, ranging from 0.7 to 29.6%. It constitutes 10 to 25% of the cases in the Gastroenterology Department, with functional constipation (FC) accounting for 90 to 95% of children’s constipation [1, 2]. FC refers to constipation that is not caused by identifiable diseases or drug factors, posing a significant impact on the physical and mental health of children, especially infants. Long-term constipation can affect their nutritional status, growth, and development [3], imposing greater economic and mental pressure on parents [4].

FC can be categorized into three types: slow transit constipation, outlet obstruction constipation, and mixed constipation. The pathogenesis of FC mainly includes insufficient intake of food fibers and water, reduced intestinal smooth muscle tension and intestinal peristalsis, mechanically obstructed intestinal peristalsis, dysfunction of defecation muscles, and disturbance of the intestinal microbiome [5, 6].

The intestinal microbiome plays an important role in the physiology and pathophysiology of constipation. It interacts with the immune system, enteric nervous system, and central nervous system, and modifies intestinal secretion and hormonal milieu. Specific microbial metabolites, such as short-chain fatty acids (SCFAs) and tryptophan catabolites, play central roles in microbiota-mediated intestinal functions [6, 7]. Recent randomized, controlled trials (RCTs) have demonstrated the anti-constipation effects of certain probiotic strains, particularly in children [8–11]. Probiotic supplementation is thus suggested as a complementary treatment for constipation.

However, the efficiency of probiotics is highly dependent on both strain specificity and disease specificity [12]. Different probiotic strains vary in efficacy due to different mechanisms-of-action against pathogens, manufacturing processes, and product quality control. Clinical guidelines and meta-analyses should recognize the importance of reporting outcomes by specific strain(s) of probiotics and the type of disease. Currently, hundreds of different probiotic products are available in the market. These products differ in excipients, amount and strains of microorganisms, and activity [13–16].

*Bifidobacterium animalis* subsp. *lactis* XLTG11 is a specific strain isolated from the intestines of healthy infants in China with independent intellectual property rights. The strain has been assigned a preservation number of CGMCC No. 18738 by the China General Microbiological Culture Collection Center (CGMCC). Worldwide, other similar probiotic strains [17–19] of *Bifidobacterium animalis* subsp. *lactis* have been shown to contribute to relieving gut dysmotility–related constipation through several mechanisms. Therefore, *Bifidobacterium animalis* subsp. *lactis* XLTG11 is also expected to have a positive effect on normalizing gut motility and maintaining gut health.

To our knowledge, no study has investigated whether XLTG11 can achieve good colonization and become a dominant microorganism to fulfill its role in gastrointestinal regulation function in children with FC. Therefore, the purpose of this research is to study the adjunctive clinical efficacy of the XLTG11 strain on FC in children.

## Materials and methods

### Subjects and ethical approval

This is a multi-center, parallel randomized, controlled, double-blinded clinical intervention. Children of both sexes and aged 0–6 years who were outpatients with FC were recruited. The study was conducted at Chengdu Women’s and Children’s Central Hospital and five sub-centers, namely Chongzhou Maternal and Child Health Care Hospital, Dayi Maternal and Child Health Care Hospital, Xindu Maternal and Child Health Care Hospital, Jinniu Maternal and Child Health Care Hospital, and Qingbaijiang Maternal and Child Health Care Hospital, spanning from November 2021 to September 2022.

The enrollment and research plan were reviewed and approved by the institutional ethics committee of the Shanghai Nutrition Society of China (ethics number of Shanghai Nutrition Society: Lun Shen [2021] No. 24). Written informed consent was obtained from each child’s parents/guardians. This study complied with the code of ethics of the World Medical Association (Declaration of Helsinki) and received approval and registration in the Chinese Clinical Trial Registration Center with the registration number ChiCTR2100053695.

### Inclusion, exclusion, and withdrawal criteria

#### ***Diagnostic criteria*** [20]*** for FC***

Must include 2 or more of the following symptoms at least once per week for a minimum of 1 month, with insufficient criteria for a diagnosis of irritable bowel syndrome:

1. Two or fewer defecations in the toilet per week in a child of a developmental age of at least 4 years.
2. At least 1 episode of fecal incontinence per week.
3. History of retentive posturing or excessive volitional stool retention.
4. History of painful or hard bowel movements.
5. Presence of a large fecal mass in the rectum.
6. History of large diameter stools that can obstruct the toilet.

After appropriate evaluation, the symptoms cannot be fully explained by another medical condition.

Inclusion criteria:

1. Conforming to Rome IV diagnostic criteria for FC in children.
2. Birth weight 2500–4000 g.
3. Age of enrollment: 0–6 years old.
4. Parents voluntarily delaying significant changes in infant feeding patterns.
5. Parents willing and able to fill in diaries and questionnaires.
6. Signed written informed consent collected from parents/guardians of enrolled children.
7. Regular and effective visits can be made during the trial.

Exclusion criteria:

1. Congenital intestinal malformation leading to outlet obstruction and difficult defecation.
2. Nervous system dysplasia and severe organic diseases, severe malnutrition, immune deficiency diseases, pancreatic dysfunction.
3. Same probiotics taken within 1 month before the diagnosis of this illness.
4. Children expected to receive antibiotic treatment during the trial.
5. Children diagnosed with allergic diarrhea.

Withdrawal criteria:

1. Erroneous inclusion and misdiagnosis.
2. Children without any clinical records for evaluation.
3. Children taking drugs prohibited by the study, including hormones, immunosuppressive drugs, and other probiotics, during the treatment.
4. Poor compliance (the number of sachets consumed was less than 80% of the expected amount).
5. Situation where the probiotic cannot be taken from the digestive tract during the treatment.
6. Children treated with antibiotics during the treatment.

### Grouping and intervention

A biostatistician, who was not directly involved in the execution of the study, used the RAND function in Excel to generate random numbers. Children who met the inclusion criteria were coded by the random numbers and assigned into the two groups based on the sequence of the random numbers. Each group was randomly assigned with 75 children.

Socio-demographic data were collected at baseline. Clinical evaluation of each child at the time of enrollment followed the Rome IV criteria [20]. Recruited children were managed per the Rome IV criteria, which included education efforts to guide families in recognizing withholding behaviors and implementing behavioral interventions such as regular toileting, maintaining diaries to monitor bowel movements, and utilizing reward systems for successful evacuations. Lifestyle counseling and dietary recommendations were developed based on the Chinese Dietary Guidelines 2022 and expert consensus and guidelines for FC in children [1, 21, 22]. The use of other laxatives, antibiotics, probiotics, fiber, fermented dairy products, and yogurt was prohibited during the study period. Glycerin suppositories were only permitted in the absence of defecation for more than 3 days.

Children in the intervention group (IG) received the oral probiotic in the form of a single sachet (SunFlower Group, Production No.: SC10632021400614). The probiotic could be taken directly or added to warm water below 45 ℃, milk, rice paste, or other liquid foods. One sachet (containing XLTG11 strain 1 × 10^10^ CFU/sachet) was taken daily for 28 consecutive days starting on the first day of the clinical treatment.

Children in the control group (CG) were treated with the placebo sachet containing only maltodextrin. The probiotic and placebo had similar appearance, taste, and smell and were provided in identical sachets with identical labeling expect for the subject specific randomization number.

If the children vomited within 30 min after taking the sachet, an additional dose was given (up to one extra dose in 4 h). Dosing and re-dosing were recorded in a case report form (CRF) by the treating physicians. The children’s parents and/or guardians, clinicians, laboratory personnel, data manager, and statistician remained blinded to group assignments until the end of data analysis.

### Data collection

After enrollment, study staff performed assessments, recorded data on CRF, and collected laboratory samples according to the protocol. Clinical medications were then assigned. During the trial period, parents took daily pictures of their child’s feces and sent them to the researcher for objective records. A fecal sample was collected daily to verify the fecal type by Bristol Stool Scale and to evaluate the treatment efficacy. The fecal consistency score, or fecal score, was evaluated based on the Bristol Stool Scale in the same order (i.e., Bristol stool type 7 had a fecal score of 7 while type 1 had a fecal score of 1). The clinicians used the CRF to record the incidence of abdominal cramps, nausea, vomiting, fever, constipation, and low appetite in children during the treatment. The mean value of weekly fecal score was defined as the sum of weekly fecal score divided by the fecal frequency in a given week.

### Fecal microbiome analysis

A total of 158 fecal samples from the children were collected for gut microbiome analyses, including 82 samples from 41 children in the IG before and after the intervention and 76 samples from 38 children in the CG. Total genome DNA from the samples was extracted by the CTAB/SDS method using a QIAamp Fast DNA fecal Mini Kit (Qiagen, Valencia, California, USA) according to the manufacturer’s instructions. The isolated genomic DNA for the bacterial 16S rRNA gene V3-V4 region was amplified using the TransGen AP221-02 Kit (TransGen, Beijing, China), with the 16S V34: 341F-806R PCR primers. The Uparse software (Uparse v7.0.1001) [23] and Quantitative Insights Into Microbial Ecology (QIIME) software [24] were utilized for 16S rRNA sequence analysis. Sequences with ≥ 97% similarity were assigned to the same operational taxonomic units (OTUs). We further selected a representative sequence for each OTU and annotated its taxonomic information based on the RDP classifier [25]. The QIIME (Version 1.9.1) calculated both alpha- (within sample) and beta- (between sample) diversity. Shannon, Simpson, Chao1, and ACE indices were used as indicators of the alpha diversity. Principal coordinate analysis (PCoA), based on Bray–Curtis distance, was used to analyze the β-diversity. The differential abundance of taxa between groups was analyzed using Kruskal–Wallis test. Differential enrichment of gut microbiota was analyzed by linear discriminant analysis effect size (LEfSe). Furthermore, LDA scores (> 4.0) derived from the LEfSe analysis at genus and species levels identified several bacterial genera and species that differed in the two groups.

To explore the functional profiles of the gut microbiota, Phylogenetic Investigation of Communities by Reconstruction of Unobserved States (PICRUSt) was performed based on 16S information from the Greengenes database [26]. The functional genes predictive analysis was performed by the NovoMagic website (https://magic.novogene.com/customer/main#/homeNew).

### Outcome measures

The primary outcome measure was the fecal frequency. Secondary outcome measures included the sum of weekly Bristol fecal score and the mean of weekly Bristol fecal score throughout the entire FC treatment episode. Moreover, adverse effects and fecal gut microbiota analysis before and after intervention were considered. Concurrently, common symptoms such as abdominal cramps, nausea, vomiting, fever, and loss of appetite were monitored throughout the entire intervention period.

### Statistical analysis

SAS version 9.2 for Windows (SAS Institute Inc., Cary, NC, USA) was used for all analyses. The quantitative data were expressed in two ways: mean ± standard deviation (SD), and median (P25, P75). *t*-test was used to compare normally distributed data. Wilcoxon rank sum test was used for data without a normal distribution. *χ*^2^ test was used to compare the difference of the treatment efficacy between the two groups for countable data. The frequency of feces, sum of weekly Bristol fecal score, and mean of weekly Bristol fecal score between the two groups before and after the intervention were compared using repeated measures analysis of variance (ANOVA). A *p*-value less than 0.05 was considered statistically significant.

### Sample size

In the present study, we considered a clinical significance to be present if the difference in weekly fecal frequency between the CG and the IG after the intervention was assumed to be more than 2 times. With a power of β = 0.8 and a significance level of α = 0.05 (bilateral), the calculated sample size for each group was approximately 60 subjects. Accounting for a 25% dropout rate, we selected a total sample size of 150 subjects with 75 subjects in each group.

## Result

### Basic clinical and demographic data

**Fig. 1:**
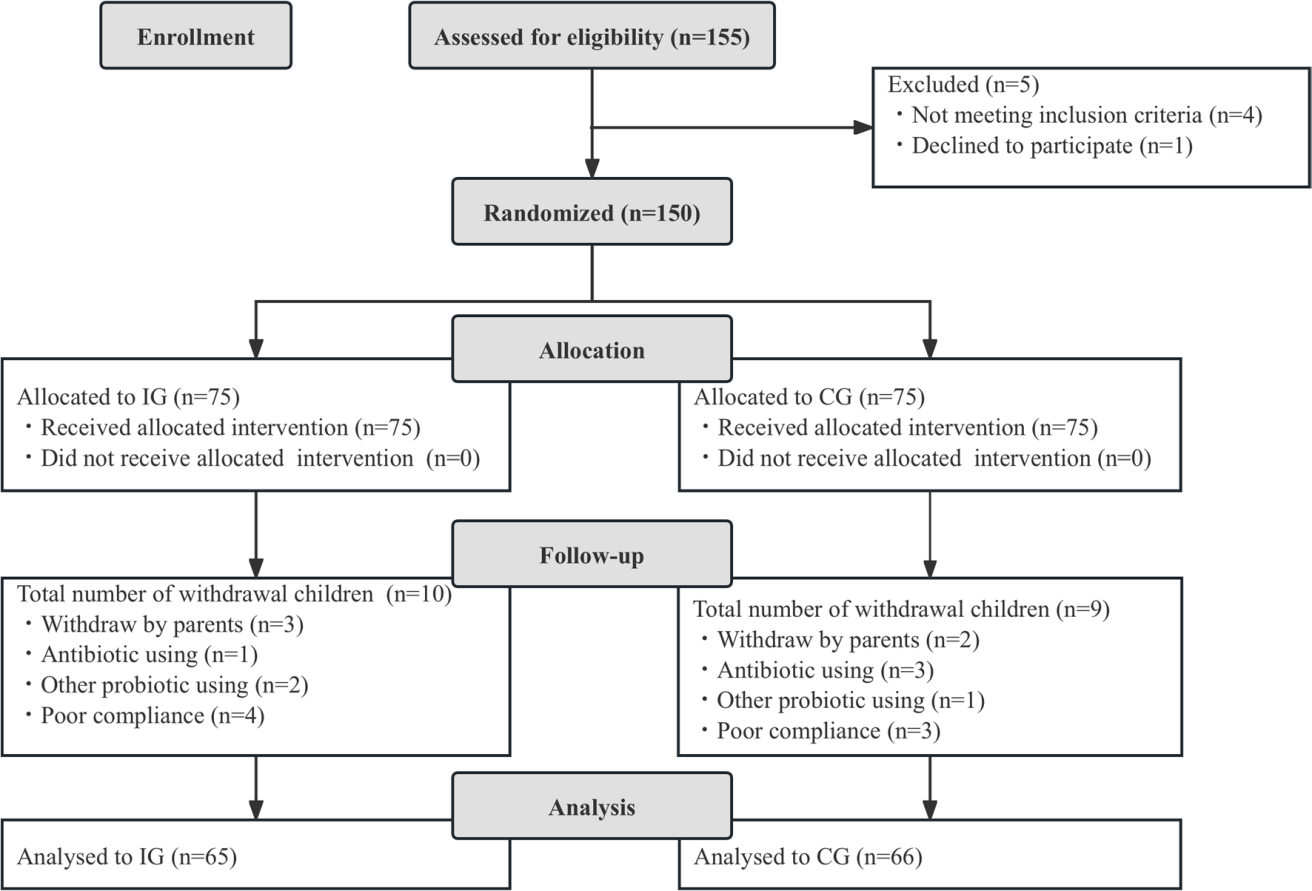
Flow chart of subject enrollment and study progress. IG, intervention group; CG, control group

A total of 150 eligible children were enrolled in the study, with approximately 12.7% (19/150) dropped out during the study period. Among these, five children were withdrawn by their parents at the initial stage, four children were treated with antibiotics during the intervention, three children took other prohibited probiotics, and seven children had poor compliance. Thus, primary and secondary outcome measures were obtained from 131 infants (65 in IG and 66 in CG, respectively) (Fig. 1).

Figure l is a flowchart illustrating participant involvement. There was no significant difference in demographics, total and mean Bristol fecal score, and weekly fecal frequency before the intervention between the two groups (*p* > 0.05, Table 1).

**Table 1:** Basic clinical and demographic data between two groups before intervention [mean ± standard deviation or median (P25, P75)]

Items		IG	CG	*χ*^*2*^ values	*p*-values
Sample size		65	66	–	–
Sex composition [males, *n* (%)]*		34 (52.31)	30 (45.45)	0.6155	0.4327
Age (month)*	Mean ± SD	20.07 ± 25.36	27.99 ± 33.83	0.8224	0.3645
	Median (P25, P75)	10.17 (6.10, 26.30)	11.88 (5.60, 32.67)		
Full term or not [yes, *n* (%)]*		52 (81.54)	61 (92.42)	3.5692	0.0589
Delivery mode [vaginal, *n* (%)]*		27 (41.54)	29 (44.62)	0.1255	0.7232
Feeding mode [n (%)]	Exclusive breast feeding	20 (30.77)	24 (36.36)	0.7446	0.6892
	Formula feeding	23 (35.38)	19 (28.79)		
	Mixed feeding	22 (33.85)	23 (34.85)		
Registered residence [urban, *n* (%)]*		44 (67.69)	47 (71.21)	0.1913	0.6619
Family history of allergic disease [yes, *n* (%)]		4 (6.15)	7 (10.61)	0.8439	0.3583
Weekly fecal frequency one week before intervention*	Mean ± SD	1.58 ± 0.56	1.46 ± 0.58	1.5510	0.2130
	Median (P25, P75)	2 (1, 2)	2 (1, 2)		
Sum of weekly Bristol fecal score one week before intervention*	Mean ± SD	2.11 ± 1.17	1.95 ± 0.98	0.1442	0.7042
	Median (P25, P75)	2 (1, 3)	2 (1, 3)		
Mean of weekly Bristol fecal score one week before intervention*	Mean ± SD	1.28 ± 0.58	1.32 ± 0.64	0.1085	0.7419
	Median (P25, P75)	1.0 (1.0, 1.5)	1.0 (1.0, 1.5)		

*There was no significant difference between the IG and the CG (*p* > 0.05)

^#^Fisher exact probability method

^+^Wilcoxon non-parametric test between groups

*IG* intervention group, *CG* control group, *SD* standard deviation

#### Effect of probiotic intervention on weekly fecal frequency

**Fig. 2:**
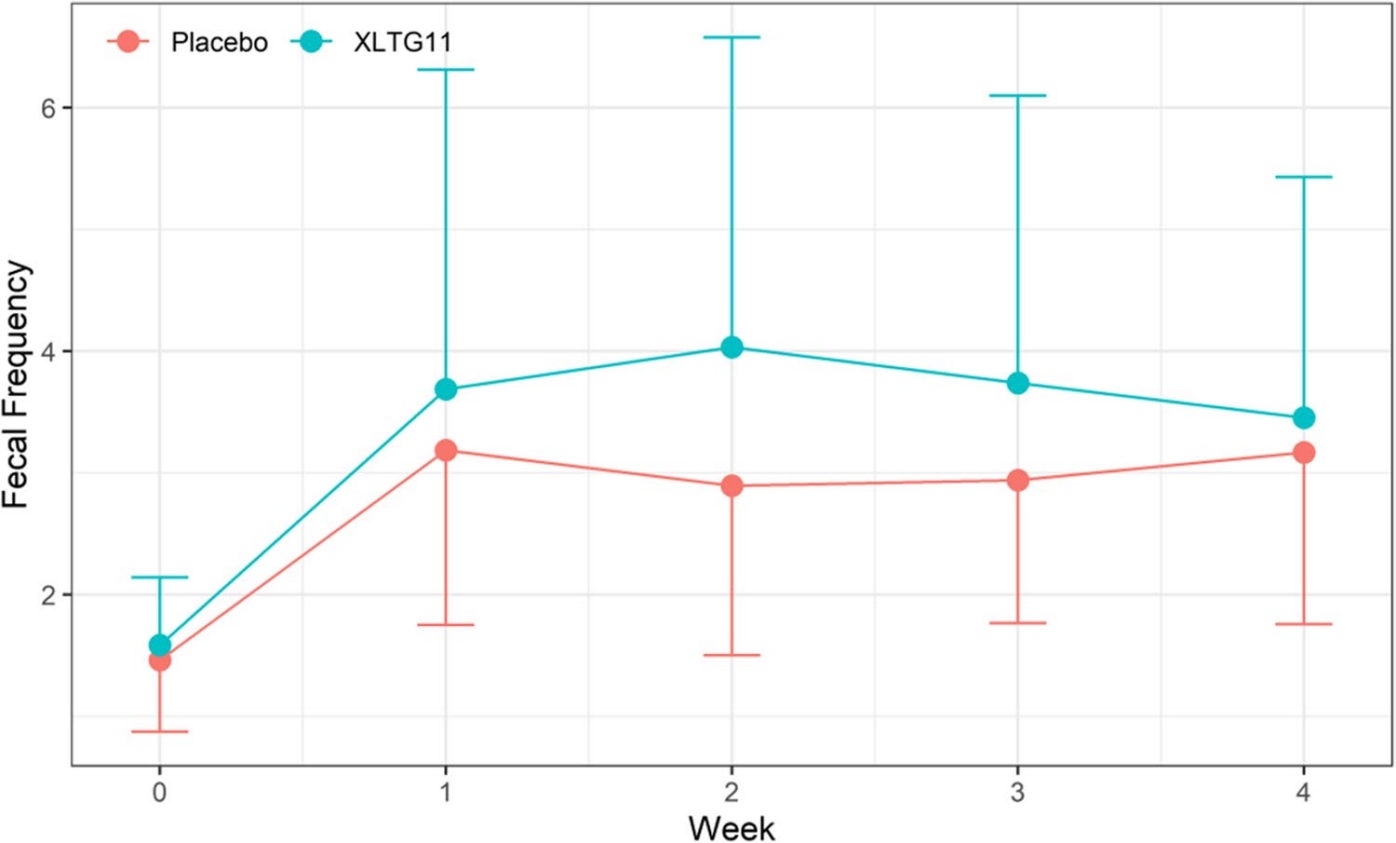
Effect of probiotic intervention on weekly fecal frequency

The weekly frequency of feces within each group increased significantly (*F* = 41.97, *p* < 0.001) with the extension of treatment time. The frequency of feces (t/w) in the IG was significantly higher than that in the CG (3.69 ± 2.62 t/w vs.3.18 ± 1.43 t/w, 4.03 ± 2.54 t/w vs. 2.89 ± 1.39 t/w and 3.74 ± 2.36 t/w vs. 2.94 ± 1.18 t/w and 3.45 ± 1.98 vs. 3.17 ± 1.41 t/w for the 1st, 2nd, 3rd, and 4th week after the intervention, respectively) (*F* = 7.60, *p* = 0.0067). However, there was no significant interaction between treatment time and intervention method (*F* = 2.11, *p* = 0.0798) (Supplementary Table 1, Fig. 2).

#### Effect of probiotic intervention on weekly fecal consistency

**Fig. 3:**
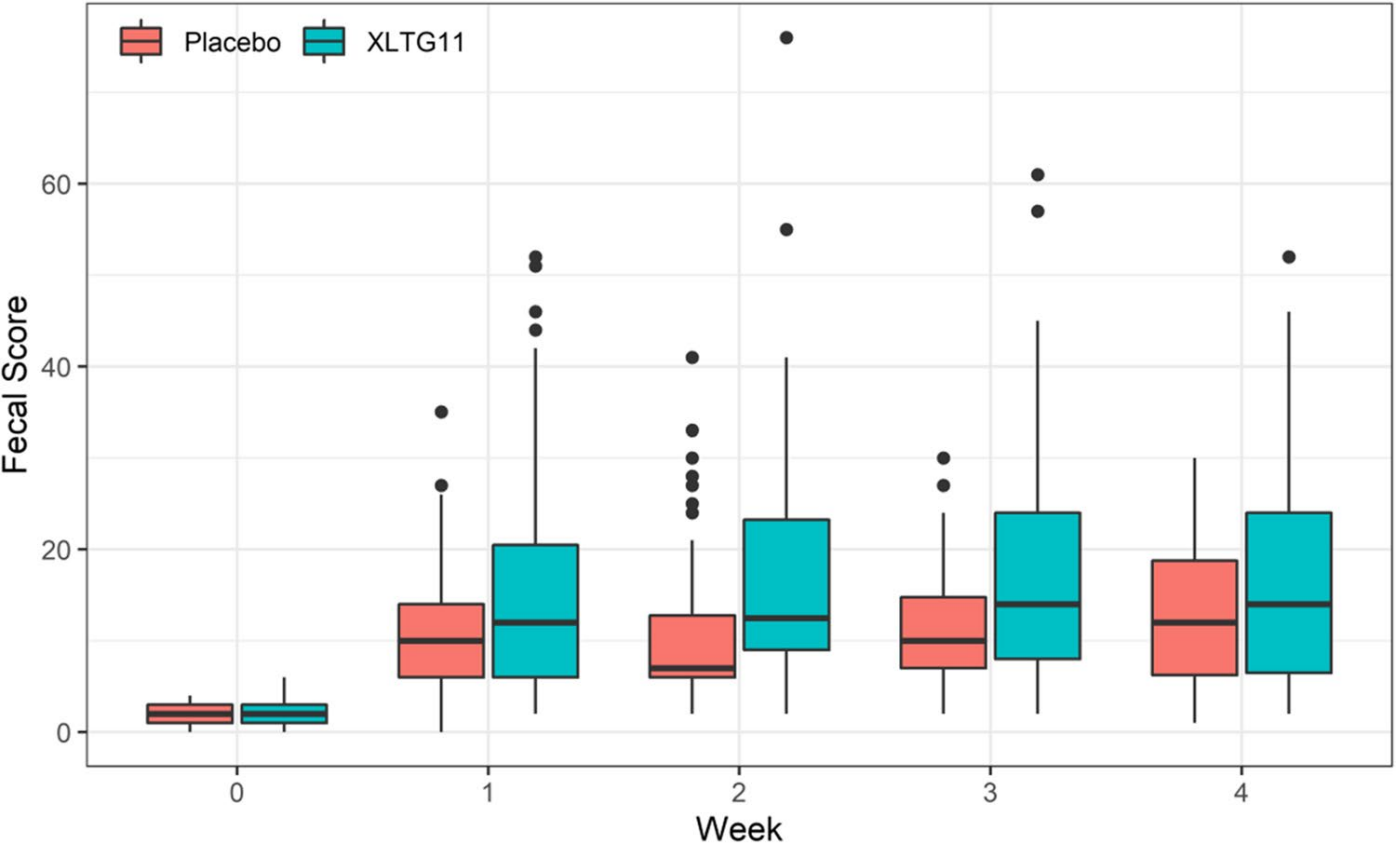
Effect of probiotic intervention on the sum of weekly Bristol fecal score

The repeated measures ANOVA indicated a significant increase in the sum of weekly Bristol fecal score for children in both groups with the extension of treatment time (*F* = 64.9, *p* < 0.001). However, children in the IG had a significantly higher sum of weekly Bristol fecal score than children in the CG (*F* = 13.94, *p* = 0.0003). There was also a significant interaction between treatment time and intervention method (*F* = 3.90, *p* = 0.0045) (Supplementary Table 2, Fig. 3).

#### Effect of probiotic intervention on the mean of weekly Bristol fecal score

**Fig. 4:**
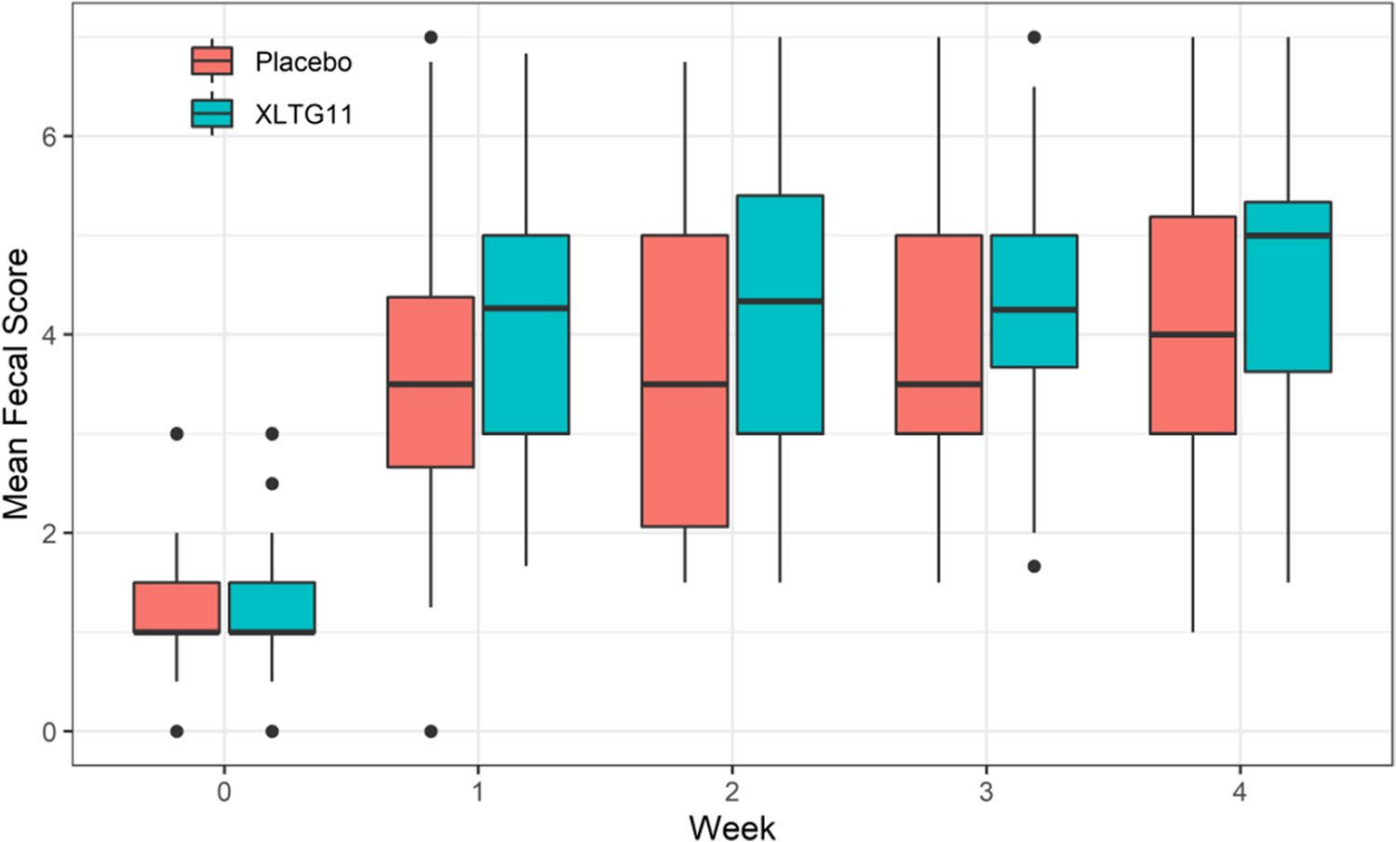
Effect of probiotic intervention on the mean of weekly Bristol fecal score

The repeated measures ANOVA showed a significant increase in the mean of weekly Bristol fecal score for children in both groups with the extension of treatment time (*F* = 197.17, *p* < 0.001). Children in the IG had significantly higher mean of weekly Bristol fecal score than children in the CG (*F* = 6.93, *p* = 0.00096). Additionally, a significant interaction between treatment time and intervention method was observed (*F* = 2.73, *p* = 0.0338) (Supplementary Table 3, Fig. 4).

#### Effect of probiotic intervention on gut microbiota

**Fig. 5:**
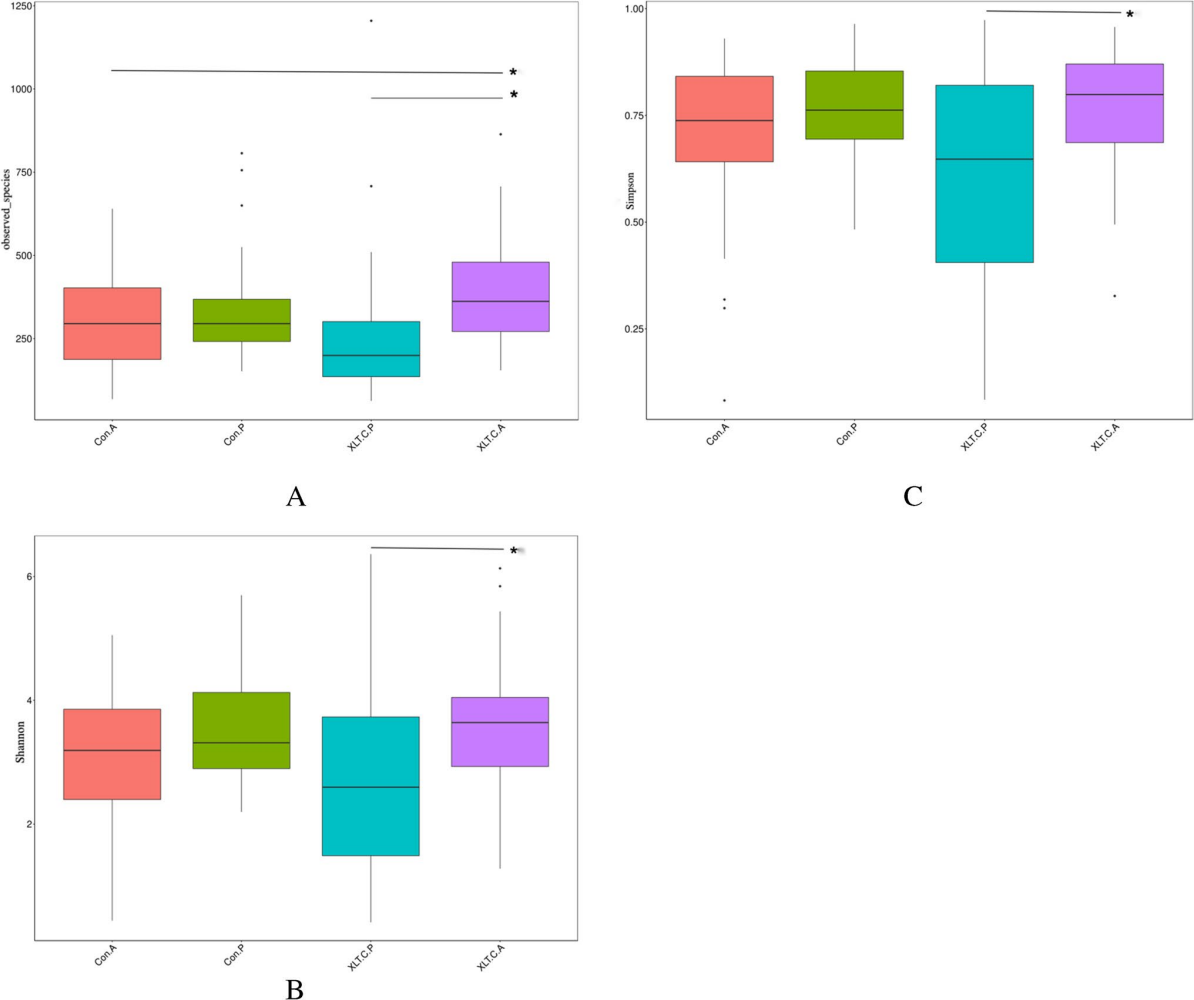
Effect of probiotic intervention on α-diversity indices of the gut microbiota. *Significant difference between groups; **A** observed species; **B** Shannon index; **C** Simpson index; Con.A, CG after intervention; Con.P, CG before intervention; XLT.C.A, IG after intervention; XLT.C.P, IG before intervention

A total of 158 fecal samples, both before and after the intervention, were collected from the IG and CG groups for the assessment of gut microbiota composition using high-throughput sequencing of the 16S rRNA gene. As shown in Fig. 5, there was no significant difference in the three alpha diversity indices between the two groups before the intervention (all *p-*values >0.05). However, after the intervention, the diversity estimates (Shannon and Simpson indices) in the IG were significantly higher than those before the intervention (all *p*<0.05). Additionally, the richness estimate indices (calculated in observed species) were significantly higher in the IG after the intervention compared to children in the CG (*p-*value <0.05).

**Fig. 6:**
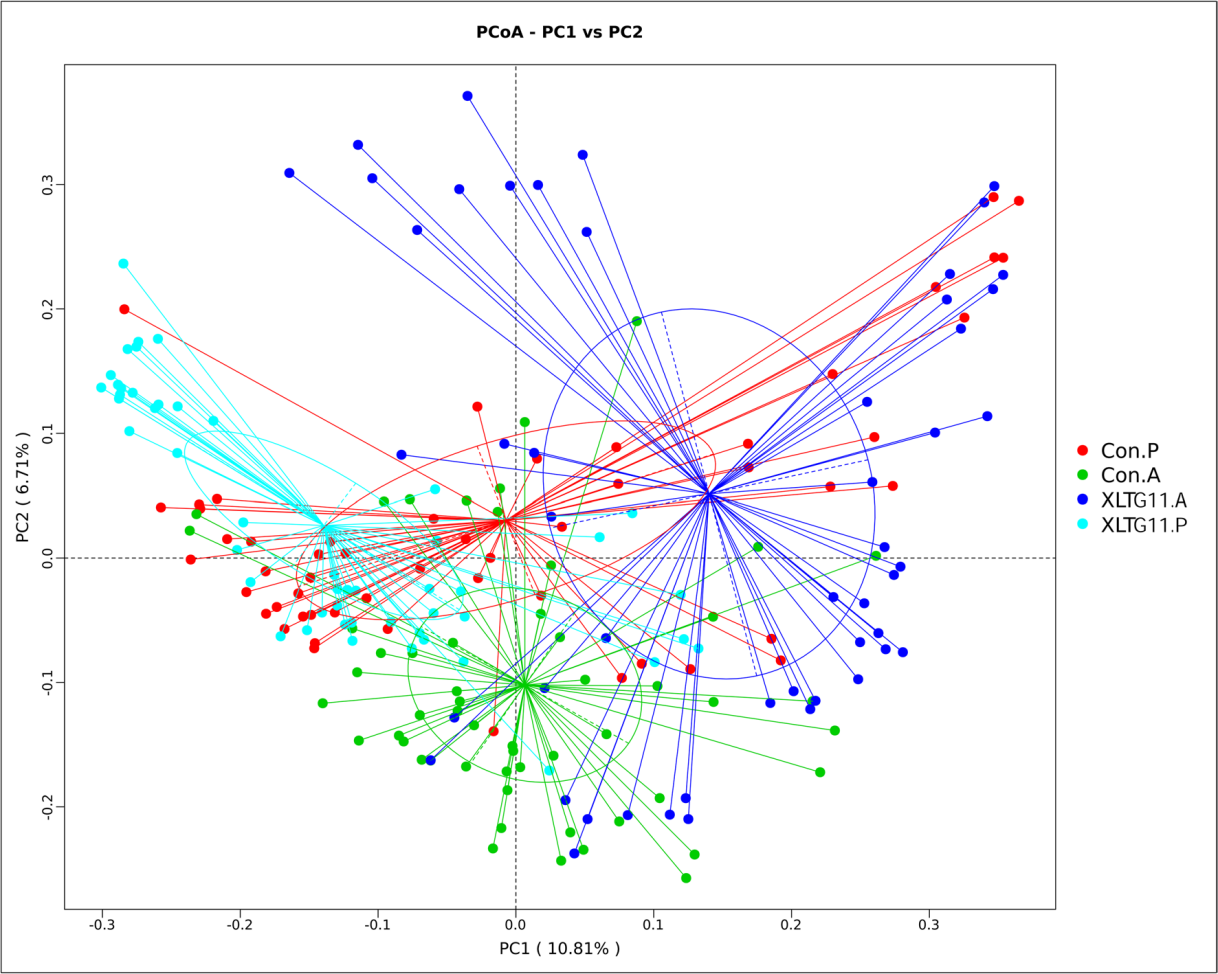
Analysis of beta diversity through principal coordinate analysis (PCoA) using Bray–Curtis distance. Con.P, control group before intervention; XLTG11.P, intervention group before intervention; Con.A, control group after intervention; XLTG11.A, intervention group after intervention

The PCoA plot based on Bray-Curtis distance showed that axis 1 (PC1) explained 10.81% of the variability, and axis 2 (PC2) explained 6.71% of the variability of before the intervention. The PCoA plot demonstrated that the samples from children in both the IG and the CG were closely situated spatially. However, after the intervention, there was a notable spatial separation between the samples from the two groups (Fig. 6).

**Fig. 7:**
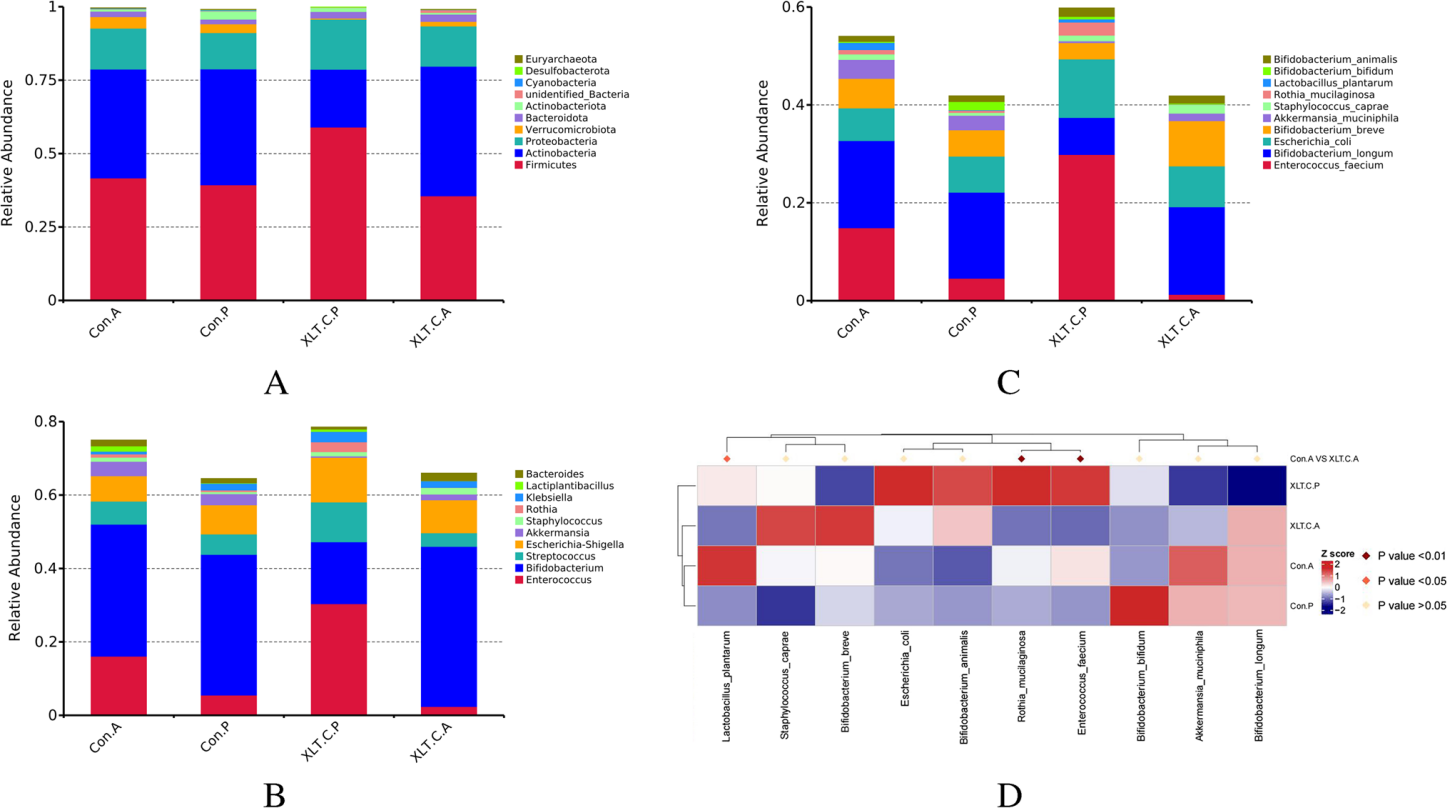
Taxa abundance at phylum (**A**), genus (**B**), and species (**C**) levels. Only the top 10 most abundant phyla, genera, and species were shown. **D** The MetaStat Complex Heat map showing the differential abundance at species level between the two groups with statistical significance. Con.A, control group after intervention; XLT.C.A, intervention group after intervention; Con.P, control group before intervention; XLT.C.P, intervention group before intervention

The gut microbiota composition is presented in Fig. 7. Before intervention, Firmicutes, Actinobacteria, and Proteobacteria dominated at the phylum level, while *Enterococcus*, *Bifidobacterium*, *Escherichia-Shigella*, and *Streptococcus* were prominent at the genus level. At the species level, *Enterococcus faecium*, *Escherichia coli*, and *Bifidobacterium longum* were predominant. After the intervention, the dominate species shifted to *Bifidobacterium longum*, *Bifidobacterium breve*, and *Escherichia coli* in the IG, while in the CG, it remained with *Enterococcus faecium*, *Bifidobacterium longum*, *Bifidobacterium breve*, and *Escherichia coli* (Fig. 7A, B, C).

**Fig. 8:**
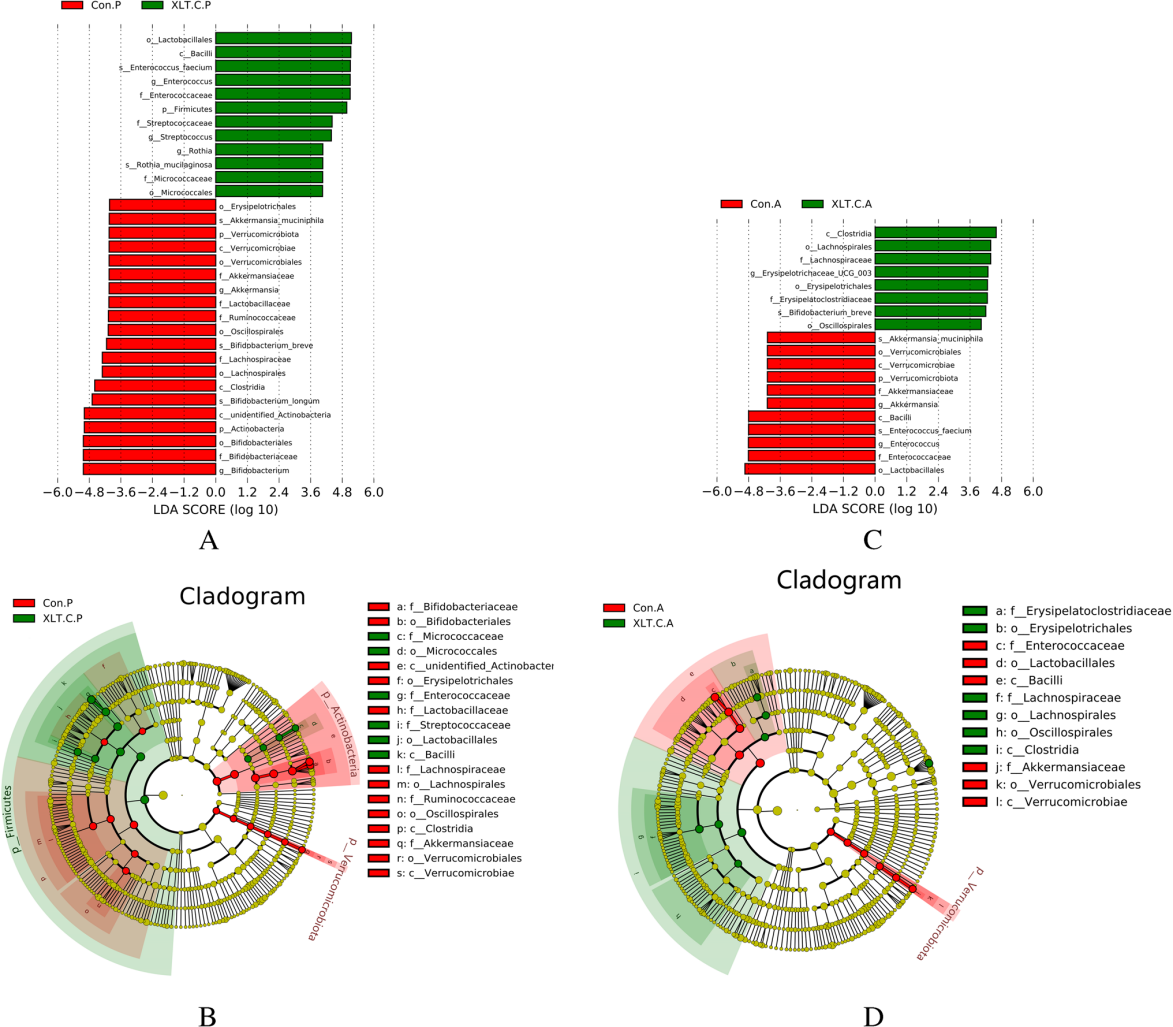
LEfSe analysis identified the most deferentially abundant taxa between the intervention and control groups. Cladogram: taxonomic representation of statistically and biologically consistent differences among intestinal microbiota of different groups. Differences were represented by the color of the most abundant taxa (green indicated a taxon with significantly higher relative abundance in the intervention group, red indicated a taxon significantly more abundant in the control group, and yellow indicated no significant difference). LAD SCORE: histogram of linear discriminant analysis (LDA) scores for deferentially abundant taxon. Cladogram was calculated by LefSe and displayed according to effect size. **A**, **B** LDA score and Cladogram before intervention; **C**, **D** LDA score and Cladogram after intervention; Con.P, control group before intervention; XLT.C.P, intervention group before intervention; Con.A, control group after intervention; XLT.C.A, intervention group after intervention

Furthermore, the MetaStat method confirmed that the abundance of *Lactobacillus plantarum*, *Rothia mucilaginosa*, and *Enterococcus faecium* in the CG was significantly higher than that in the IG (*p*<0.05) (Fig. 8D).

To compare the differences in gut microbiota community composition between the IG and CG, LEfSe analysis was performed. LEfSe analysis identified 19 taxa that exhibited differential abundance between the two groups before the intervention. Following the intervention, 12 taxa showed differential abundance (Fig. 8B, D). In comparison to the CG, XLTG11 treatment increased abundance of two families (Erysipelatoclostridiaceae and Lachnospiraceae), three orders (Erysipelotrichales, Lachnospirales, and Oscillospirales), and one class (Clostridia).

According to LDA scores, notable high abundance was observed in the *Bifidobacterium breve* species and *Erysipelotrichaceae_UCG_003* genus in children from the IG, while the children in the CG showed enrichment with the *Akkermansia muciniphila* and *Enterococcus faecium* species, and the *Akkermansia* and *Enterococcus* genera after the intervention (Fig. 8A, B).

#### XLTG11 treatment changed the functional gene composition of gut microbiota

**Fig. 9:**
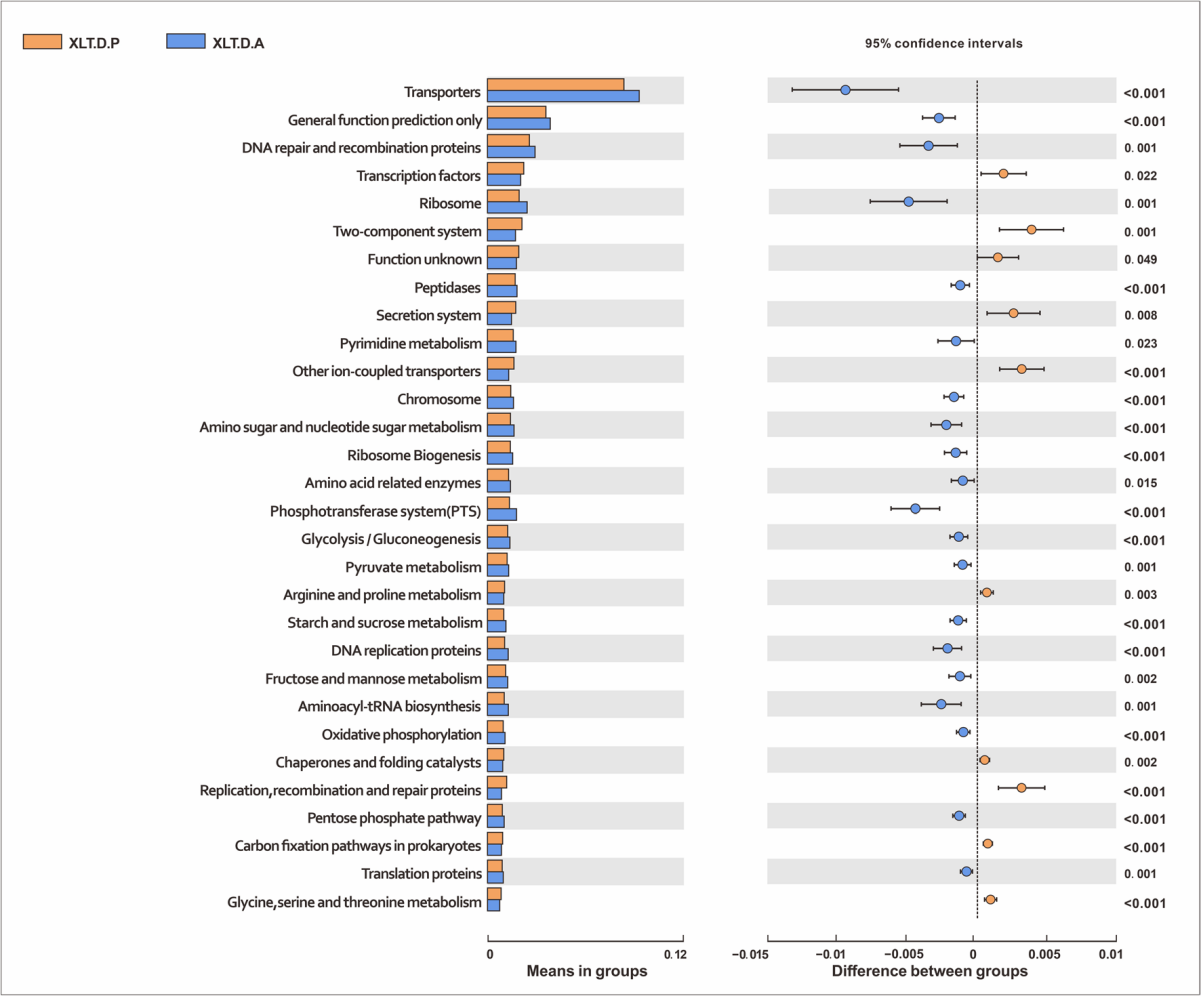
PICRUSt function prediction of the gut microbiota in the XLTG11 group with top 30 means (Welch’s *t* test, two-sided, *p* < 0.05) XLT.C.A, intervention group after intervention; XLT.C.P, intervention group before intervention

To explore the effects of XLTG11 intervention on physiological functions, PICRUSt was used to analyze and predict the composition of the functional genes in the metabolic pathways. The result showed a significant difference in functional genes within the gut microbiota before and after XLTG11 treatment, suggesting a potential impact on the metabolic pathways of the gut microbiota. Notably, the proportion of 50 sub-functional genes in the gut microbiota exhibited evident changes after XLTG11 treatment (Fig. 9). Before XLTG11 treatment, functional genes involved in peptidases, chromosome, amino acid–related enzymes, ribosome biogenesis, arginine and proline metabolism, methane metabolism, and DNA replication proteins were upregulated. Conversely, after XLTG11 treatment, different functional genes such as pyruvate metabolism, bacterial motility proteins, butanoate metabolism, propanoate metabolism, citrate cycle, lipid biosynthesis proteins, tyrosine metabolism, and fatty acid metabolism were upregulated. These findings indicated a significant influence of XLTG11 treatment on the composition of functional genes within the gut microbiota.

#### Incidence of probiotic intervention related adverse reactions during treatment

No incidence of abdominal colic, nausea, vomiting, fever, low appetite, and other symptoms related to probiotic intervention was reported in both groups.

## Discussion

*Bifidobacterium animalis* subsp. *lactis* XLTG11 is a specific strain isolated from the intestines of healthy infants. It demonstrates good gastrointestinal passage abilities and strong antibacterial effect against common human pathogens, such as *Escherichia coli*, *Salmonella*, and *Staphylococcus aureus*. Animal studies and in vitro research have found that XLTG11 can alleviate DSS-induced colitis and ameliorate antibiotic-related diarrhea by inhibiting the activation of the TLR4/MYD88/NF-κB signaling pathway, regulating inflammatory cytokines, improving intestinal barrier function, and modulating gut microbiota composition and intestinal immunity [27–29].

As an auxiliary treatment, the use of XLTG11 independently demonstrates effective improvement in FC in children, starting from the first week of treatment. This finding aligns with a recent study in China that used *Bifidobacterium lactis* Bb-12 strain for treating FC in infants [30]. In addition, a recent meta-analysis on probiotics for children with FC also indicated [31] that probiotic use can increase fecal frequency. However, conflicting results suggesting ineffectiveness have been reported in other studies [32]. Considering the specificity of the strains, we believe that this “ineffectiveness” may be attributed to the specific strains used in these studies and the heterogeneity in research subjects, and may not extend to all other probiotics.

The increased fecal frequency may be attributed to two factors: the time effect and the intervention effect. The weekly fecal frequency in both groups significantly increased with the extension of treatment time, indicating the effectiveness of the treatment protocols in both groups. Additionally, the probiotic intervention exhibited additional treatment effects in alleviating FC after accounting for the time effect. However, no interaction between the time effect and intervention effect was found. Besides the increased fecal frequency, the probiotic significantly improved the fecal consistency in children with FC. Nevertheless, in contrast to the changes in fecal frequency, the interaction effect indicated that with the extension of treatment time, the improvement in fecal consistency in the probiotic intervention group was more pronounced.

The alteration in gut microbiota composition within the intestinal micro-ecology and the influence of probiotics on this micro-ecology are closely related to the therapeutic effect and clinical progression of gastrointestinal diseases. Recent studies investigating the use of *Bifidobacterium animalis* as an adjuvant therapy for gastrointestinal diseases in both children and adults have shown that such usage can regulate the composition of intestinal micro-ecology and enhance prognosis. Examples include *Bifidobacterium animalis* subsp. *lactis* BB-12 for infants with colic [33] and individuals receiving antibiotics [34], *Bifidobacterium lactis* Probio-M8 for asthmatic patients [35], and *Bifidobacterium animalis* subsp. *lactis* BL04 for microbiota regulation in healthy adults [36]. The findings of the present study align closely with those mentioned above. Although both groups of children with FC achieved clinical cure outcomes through different treatment methods, the recovery duration and the dynamics of changes in fecal frequency and consistency were not entirely similar. This discrepancy might be partially attributed to distinct alterations in the intestinal microbiome observed in the two groups of children.

In our study, it is reasonable to infer that the intestinal immune function and the incidence rate of gastrointestinal diseases during the follow-up of children in the IG will likely be superior to those of children in the CG due to the distinct changes in gut microbiota composition after intervention. This inference can be validated through the long-term follow-up of participating children. Considering the high diversity of gut microbiota of infants as a characteristic following probiotic administration and the reduced risk of gastrointestinal diseases due to increased variety and abundance of dominant or beneficial bacteria, the high diversity of gut microbiota after the use of XLTG11 might be a manifestation of the microbiota regulation of XLTG11. Although infants in the CG also experienced recovery from constipation after treatment, their gut microbiota composition differed significantly from that of infants in the IG. Not only did the diversity of gut microbiota increase, but the abundance of some potential pathogens also increased. Whether the different gut microbiota composition between the two groups will affect the incidence rate of diarrhea in a later period warrants a long-term follow-up study.

According to the functional gene prediction analysis, XLTG11 treatment might upregulate functional genes involved in short-chain fatty acid (SCFA) metabolism. SCFAs are major fermentation products of the gut microbiota [37]. They may play a role in the pathogenesis of FC by regulating colonic electrolyte absorption and secretion, mucin secretion, and colonic motility. Previous studies on a similar strain, *Bifidobacterium animalis* subsp. *lactis* HN019, have demonstrated its ability to reduce intestinal transit time and increase bowel movement frequency in FC, possibly through SCFAs derived from microbial fermentation [38]. Therefore, the upregulated SCFAs metabolism could contribute to the amelioration of FC by regulating intestinal function. Moreover, bacterial motility is facilitated in most bacterial species through the flagellar apparatus [39], which consists of numerous bacterial motility proteins with thousands of individual subunits. Notably, bacterial flagella are potent inflammatory structures [40]. Hence, alterations in the functional predicted genes related to bacterial motility proteins could play a role in alleviating the symptoms experienced by patients with FC. It is also worth noting that after XLTG11 treatment, the functional gene prediction of methane metabolism was downregulated. Methane has been implicated as a factor that decreases ileal and colon transit time, raises the amplitude of contraction, slows peristalsis, and leads to constipation [41]. Studies have shown that targeting methanogenesis directly can relieve symptoms in constipation-predominant irritable bowel syndrome [41]. Therefore, it is reasonable to infer that one possible mechanism of XLTG11 in improving constipation symptoms might be related to its influence on intestinal methanogenesis. However, further research is needed to elucidate the role of XLTG11 in methanogenesis. While these results suggest that XLTG11 treatment might regulate SCFAs and methane metabolism–related genes in the gut microbiota to alleviate FC symptoms, additional clinical trials, animal studies, and in vitro research are necessary to confirm this conclusion.

Mechanistic research on the impact of probiotics on FC have showed that specific probiotics can influence microbiota composition, SCFA production, inflammatory cytokines, bile acid metabolism, the mucus layer, and components of the immune system that could potentially affect gut motility [6]. This study provides a preliminary demonstration that XLTG11 may, at least partially, affect the adjuvant treatment efficiency and long-term prognosis of constipation through these mechanisms. However, further research is required to comprehensively understand the intricate interactions involving specific strain like XLTG11 and constipation.

### Limitation analysis

Firstly, the use of a single dose of XLTG11 at 1 × 10^10^ CFU/day limits our ability to investigate the optimal dose–response relationship of XLTG11 strain in the adjuvant treatment of FC. Secondly, due to the limitations of the study’s primary objectives and design, the probiotic intervention and clinical symptom observation were restricted to a duration of only 4 weeks. Therefore, the potential long-term effects of XLTG11 on children’s health and gut microbiota cannot be adequately observed. Lastly, the effect of exogenous probiotic supplementation on intestinal function is influenced by various bias factors, such as the mode of delivery, feeding method, and allergic disease. Although some of these factors have been considered, it is possible that the intervention effect of XLTG11 may be compromised. Future studies could benefit from expanding the sample size and extending the observation time to enhance the reliability and representativeness of the research conclusions. Additionally, further exploration of the long-term effects of XLTG11 on children’s health would be valuable.

## Conclusions

In summary, no adverse effects of XLTG11 intervention were observed during our study period, indicating the safety of XLTG11 for children. The administration of *Bifidobacterium animalis* subsp. *lactis* XLTG11 at a dose of 1 × 10^10^ CFU/day to children aged 0–6 years can increase fecal frequency, improve fecal consistency, induce beneficial changes in intestinal microbiota composition, and regulate SCFs and methane metabolism-related genes in the gut microbiota of children with FC.

### Supplementary Information

Below is the link to the electronic supplementary material.

Supplementary file1 (DOCX 17 KB)

## Data Availability

The data are available from the corresponding author on reasonable request.
