# Supplementary material for: Adjunctive efficacy of Bifidobacterium animalis subsp. lactis XLTG11 for functional constipation in children
Source: Braz J Microbiol. 2024 Feb 21;55(2):1317–30. doi: 10.1007/s42770-024-01276-3 (PMC11153453; doi:10.1007/s42770-024-01276-3)
Supplement: Supplementary file 1 — Supplementary file1 (DOCX 17 KB) [file 42770_2024_1276_MOESM1_ESM.docx]

Supplementary Tables

Supplementary Table 1 Efficiency of probiotic intervention on weekly fecal frequency of children between two groups [mean ± standard deviation, median (P25, p75)]

| Weekly fecal frequency | | | IG (n=65) | CG (n=66) |
| --- | --- | --- | --- | --- |
| one week before intervention | | mean±SD | 1.58±0.56 | 1.46±0.58 |
|  |  | median(P25,P75) | 2(1, 2) | 2(1, 2) |
| 1st week during intervention | | mean±SD | 3.69±2.62 | 3.18±1.43 |
|  |  | median(P25,P75) | 3(2, 5) | 3(2, 4) |
| 2nd week during intervention | | mean±SD | 4.03±2.54 | 2.89±1.39 |
|  |  | median(P25,P75) | 3(2, 5) | 3(2, 3) |
| 3rd week during intervention | | mean±SD | 3.74±2.36 | 2.94±1.18 |
|  |  | median(P25,P75) | 3(2, 5) | 3(2, 4) |
| 4th week during intervention | | mean±SD | 3.45±1.98 | 3.17±1.41 |
|  |  | median(P25,P75) | 3(2, 4) | 3(2, 4) |
| Time efficiency | *F* value^*^ | 41.97 | | |
|  | *p* value | <0.0001 | | |
| Intervention efficiency | *F* value^*^ | 7.60 | | |
|  | *p* value | 0.0067 | | |
| Time-intervention interaction efficiency | *F* value^*^ | 2.11 | | |
|  | *p* value | 0.0798^**^ | | |

*, analysis of variance of repeated measurement data; IG, intervention group; CG, control group; SD, standard deviation; **, post-hoc analysis.

Supplementary Table 2 Efficiency of probiotic intervention on the sum of weekly Bristol fecal score of children between the two groups [mean ± standard deviation, median (P25, p75)]

| Sum of daily Bristol fecal score | | | IG (n=65) | CG (n=66) |
| --- | --- | --- | --- | --- |
| one week before intervention | | mean±SD | 2.11±1.17 | 1.95±0.98 |
|  |  | median(P25, P75) | 2(1, 3) | 2(1, 3) |
| 1st week during intervention | | mean±SD | 16.34±13.97 | 10.6±6.54 |
|  |  | median(P25, P75) | 12(6, 21) | 10(6, 14) |
| 2nd week during intervention | | mean±SD | 17.56±13.51 | 11±8.43 |
|  |  | median(P25, P75) | 12.5(9.0, 23.5) | 7(6, 13) |
| 3rd week during intervention | | mean±SD | 17±12.74 | 11.58±6.16 |
|  |  | median(P25, P75) | 14(8, 24) | 10(7, 15) |
| 4th week during intervention | | mean±SD | 16.21±10.89 | 13.29±7.97 |
|  |  | median(P25, P75) | 14(6, 24) | 12(6, 19) |
| Time efficiency | *F* value^*^ | 64.91 | | |
|  | *p* value | <0.0001 | | |
| Intervention efficiency | *F* value^*^ | 13.94 | | |
|  | *p* value | 0.0003 | | |
| Time-intervention interaction efficiency | *F* value^*^ | 3.90 | | |
|  | *p* value | 0.0045^**^ | | |

*, Analysis of variance of repeated measurement data; IG, intervention group; CG, control group; SD, standard deviation; **, post-hoc analysis.

Supplementary Table 3 Efficiency of probiotic intervention on the mean of weekly Bristol fecal score of children between the two groups [mean ± standard deviation, median (P25, p75)]

| Mean of daily Bristol fecal score | | | IG (n=65) | CG (n=66) |
| --- | --- | --- | --- | --- |
| one week before intervention | | mean±SD | 1.28±0.58 | 1.32±0.64 |
|  |  | median(P25, P75) | 1.0(1.0, 1.5) | 1.0(1.0, 1.5) |
| 1st week during intervention | | mean±SD | 4.18±1.36 | 3.60±1.43 |
|  |  | median(P25, P75) | 4.27(3.0, 5.0) | 3.5(2.67, 4.38) |
| 2nd week during intervention | | mean±SD | 4.28±1.33 | 3.64±1.56 |
|  |  | median(P25, P75) | 4.33(3.0, 5.4) | 3.5(2.0, 5.0) |
| 3rd week during intervention | | mean±SD | 4.34±1.30 | 3.94±1.40 |
|  |  | median(P25, P75) | 4.25(3.67, 5.0) | 3.5(3.0, 5.0) |
| 4th week during intervention | | mean±SD | 4.49±1.29 | 4.05±1.45 |
|  |  | median(P25, P75) | 5.0(3.50, 5.33) | 4.0(3.0, 5.25) |
| Time efficiency | *F* value^*^ | 197.17 | | |
|  | *p* value | <0.0001 | | |
| Intervention efficiency | *F* value^*^ | 6.93 | | |
|  | *p* value | 0.0096 | | |
| Time-intervention interaction efficiency | *F* value^*^ | 2.73 | | |
|  | *p* value | 0.0338^**^ | | |

*, Analysis of variance of repeated measurement data; IG, intervention group; CG, control group; SD, standard deviation; **, post-hoc analysis.
